# Supplementary material for: Gut Microbiota across Normal Gestation and Gestational Diabetes Mellitus: A Cohort Analysis
Source: Metabolites. 2022 Aug 26;12(9):796. doi: 10.3390/metabo12090796 (PMC9504460; doi:10.3390/metabo12090796)
Supplement: Supplementary file 1 [file metabolites-12-00796-s001.zip › metabolites-1880427-supplementary.pdf]

Figure S1. Correlation between bacteria and food intake in first/second trimester (Panel A) and third trimester (Panel B)

Panel A

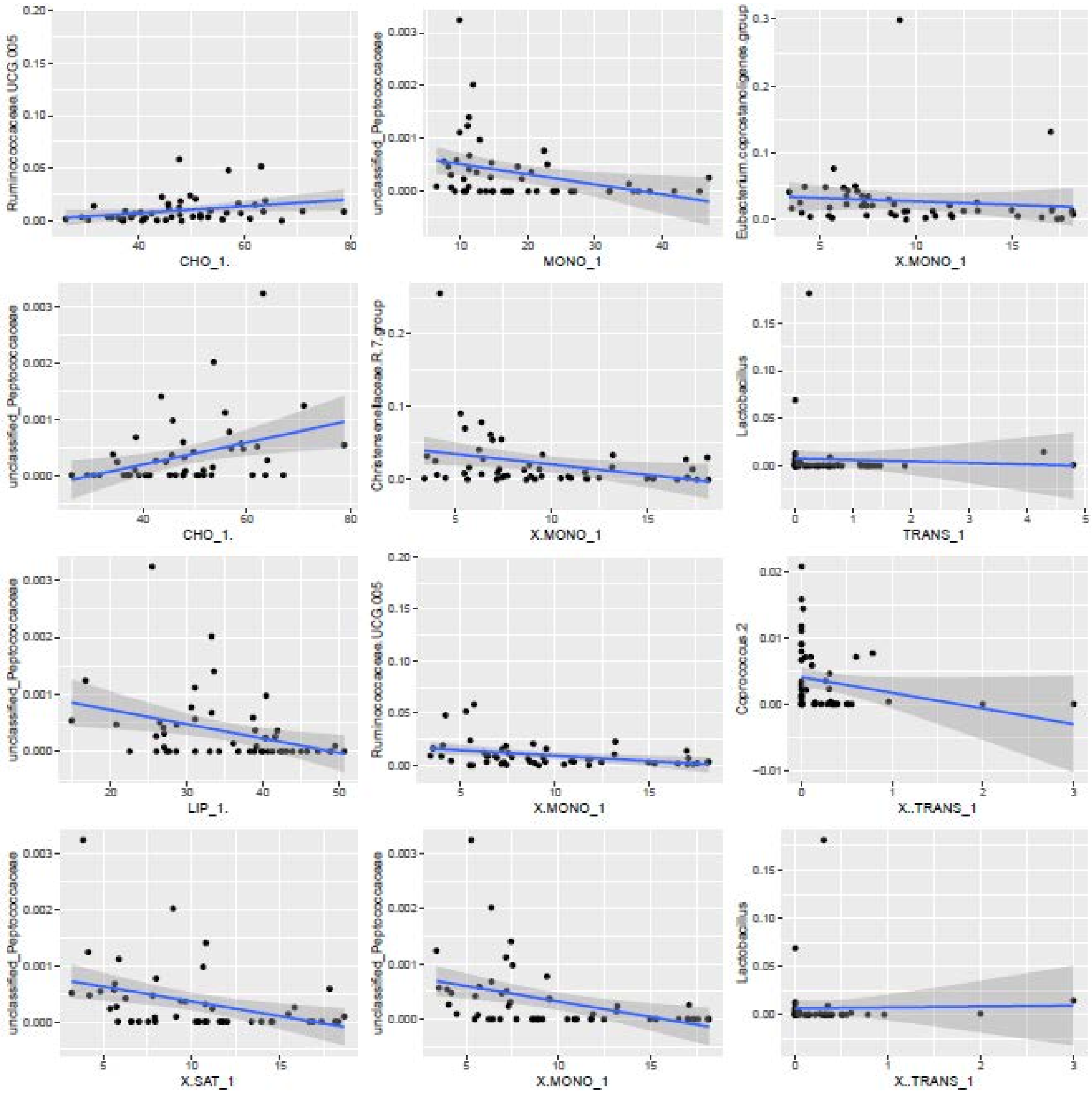

Panel B

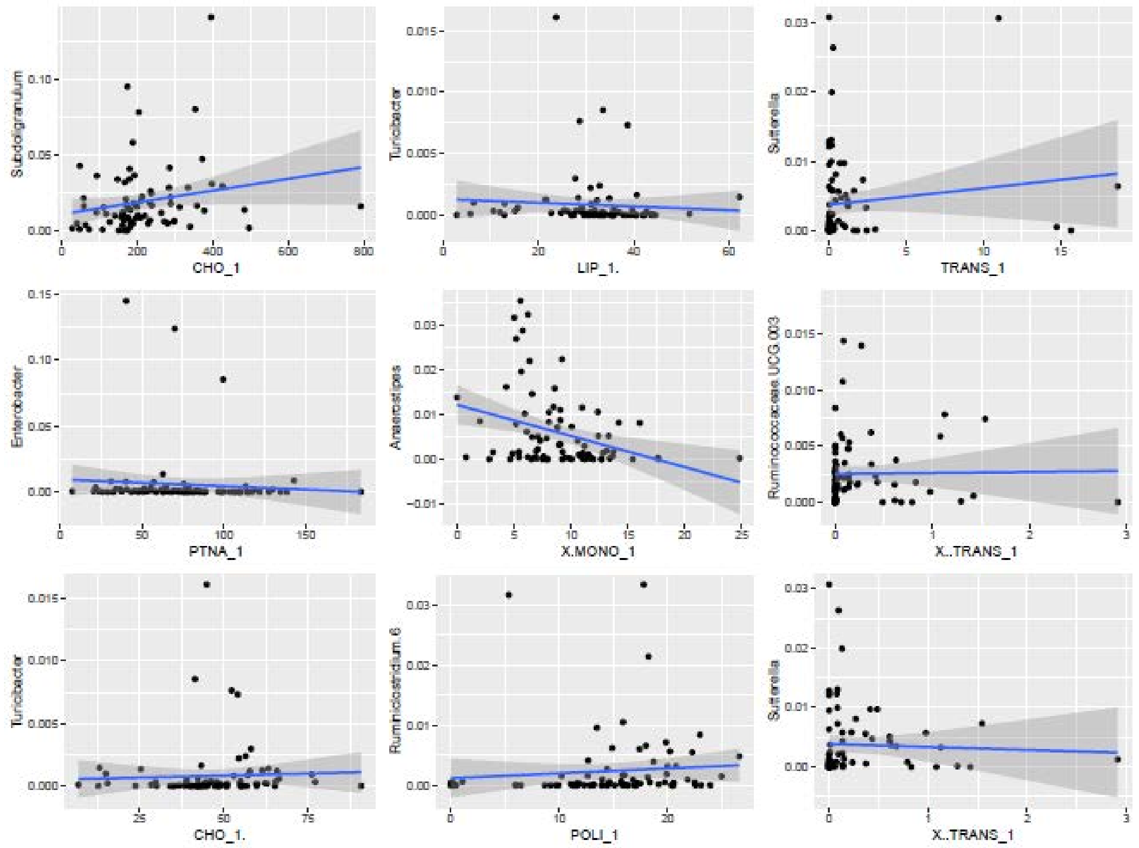
Subdoligranulum
CHO_1
Turicibacter
LIP_1.
Sutterella
TRANS_1
Enterobacter
PTNA_1
Anaerostipes
X.MONO_1
Ruminococcaceae.UCG.003
X..TRANS_1
Turicibacter
CHO_1.
Ruminiclostridium.6
POLI_1
Sutterella
X..TRANS_1
